# The Role of Selected Bioactive Compounds in the Prevention of Alzheimer’s Disease

**DOI:** 10.3390/antiox9030229

**Published:** 2020-03-11

**Authors:** Wojciech Grodzicki, Katarzyna Dziendzikowska

**Affiliations:** 1 Faculty of Human Nutrition, Warsaw University of Life Sciences, Nowoursynowska 159c, 02-776 Warsaw, Poland; wojciech_grodzicki@mail.sggw.pl; 2 Department of Dietetics, Institute of Human Nutrition Sciences, Warsaw University of Life Sciences, Nowoursynowska 159c, 02-776 Warsaw, Poland

**Keywords:** neurodegeneration, Alzheimer’s disease, neuroprotection, bioactive compounds, MIND diet

## Abstract

Neurodegeneration is a feature of many debilitating, incurable age-dependent diseases that affect the nervous system and represent a major threat to the health of elderly persons. Because of the ongoing process of aging experienced by modern societies, the increasing prevalence of neurodegenerative diseases is becoming a global public health concern. A major cause of age-related dementia is Alzheimer’s disease (AD). Currently, there are no effective therapies to slow, stop, or reverse the progression of this disease. However, many studies have suggested that modification of lifestyle factors, such as the introduction of an appropriate diet, can delay or prevent the onset of this disorder. Diet is currently considered to be a crucial factor in controlling health and protecting oneself against oxidative stress and chronic inflammation, and thus against chronic degenerative diseases. A large number of bioactive food compounds may influence the pathological mechanisms underlying AD. Among them, phenolic compounds, omega-3 fatty acids, fat-soluble vitamins, isothiocyanates, and carotenoids seem to be promising. They act not only as antioxidant and anti-inflammatory agents, but also as active modulators of the pathological molecular mechanisms that play a role in AD development, including the formation of amyloid plaques and tau tangles, the main hallmarks of AD pathology. In vivo animal model studies as well as clinical and epidemiological research suggest that nutritional intervention has a positive effect on the health of older people and may prevent age-related cognitive decline, especially when the diet contains more than one bioactive nutrient. The Mediterranean diet and in particular its combination with Dietary Approaches to Stop Hypertension, which is called the MIND diet, are nutritional patterns based on many products rich in bioactive compounds that appear to be the most effective in preventing neurodegeneration. The present review gathers evidence that supports the neuroprotective effect of bioactive substances.

## 1. Introduction

Neurodegenerative diseases constitute one of the most urgent health concerns in developed countries. Because their progression usually lasts for decades and the first symptoms tend to appear late in life, the ongoing process of aging experienced by modern societies contributes to the rising prevalence of these disorders [1]. Therefore, a significant part of the research effort has shifted toward preventive methods. A growing body of evidence suggests that a healthy lifestyle, including a balanced diet rich in bioactive compounds, can provide neuroprotection and reduce the risk of central nervous system (CNS) pathologies [2].

The most common neurodegenerative disorder is Alzheimer’s disease (AD). It accounts for 60%–80% of all cases of dementia, which is the fifth leading cause of death worldwide [2,3]. AD affects primarily the elderly population and is becoming a global health concern; in 2015, 47 million people suffered from dementia, and this number is estimated to reach 131 million in 2050 [4]. Currently, there are no effective therapies to stop or reverse the disease; therefore, a lot of studies are focused on preventive methods for AD. Nutrition is one of the primary lifestyle factors influencing the risk of AD, and previous research has indicated that its preventive potential can be related to bioactive compounds derived from different food products [5]. Several studies have suggested that healthy nutrition is one of the primary lifestyle factors that could reduce the risk of AD [6,7,8]. A well-balanced diet can provide neuroprotection, which indicates that bioactive compounds could influence the underlying pathological mechanisms of AD [5,7,9,10].

## 2. Alzheimer’s Disease: Etiology and Pathophysiology 

Although AD was reported for the first time over 100 years ago by a German doctor, Aloise Alzheimer, the underlying mechanisms responsible for the development of AD yet remain unclear [11]. Characteristic symptoms of AD include dementia, memory and spatial awareness impairment, movement dysfunction, depression, delusion, and hallucination. Patients often experience anomic aphasia, acalculia, and apathy. During the final stage of the disease, patients cannot communicate verbally, lose independency, and are unable to perform basic daily functions [1,12].

Behavioral changes observed in AD are manifestations of the underlying processes affecting the CNS. Despite the continuous research effort, the etiopathogenesis of this disorder has not yet been fully explained. However, some characteristic mechanisms have been identified at both cellular and tissue levels. A typical process observed in AD is the accumulation of amyloid beta (Aβ). Aβ forms aggregates known as senile plaques and is a short peptide produced from naturally occurring amyloid precursor protein (APP). Under physiological conditions, Aβ performs important regulatory functions, plays a role in axonal growth, and influences synaptic plasticity [12,13].

**Figure 1:**
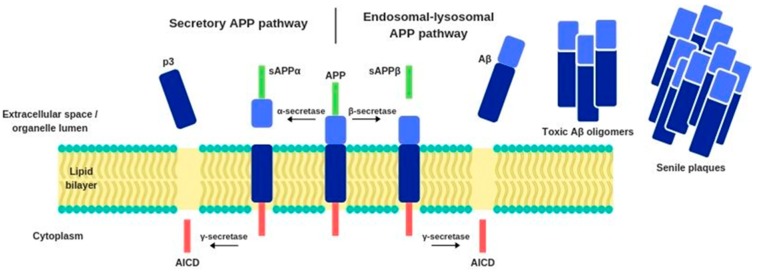
Biochemical pathways of APP modification.

Under normal conditions, the APP is metabolized through the secretory pathway, which consists of its transport from the endoplasmic reticulum to the Golgi apparatus and further to the cellular membrane, where its proteolytic modifications occur. The reaction is catalyzed by the enzyme α-secretase, which cleaves APP into a soluble sAPPα protein and another fragment that is further proteolyzed by γ-secretase. This process generates an intracellular APP domain (AICD) and a p3 fragment. However, under pathological conditions, APP can enter an alternative endosomal-lysosomal APP proteolytic processing pathway. APP biochemical modifications occur also under the influence of β-secretase, which operates closer to the APP N-terminus, near to the lumen of the cellular organelles, while the γ-secretase works near the C-terminus of APP, which is immersed in the cytoplasm. β-secretase enzyme’s catalytic activity generates a sAPPβ protein. This process produces not only AICD but also another insoluble peptide—amyloid beta (Figure 1). Compared to healthy individuals, the alternative APP cleavage by β-secretase is 50% more frequent in AD patients. Consequently, there is a high amount of Aβ in the extracellular space, where it binds to apolipoprotein E (*APOE*), degenerated axons, microglia, and astrocytes activated by proinflammatory cytokines [12,13,14,15].

Senile plaques can penetrate the blood vessels and disrupt the blood supply to the brain. Furthermore, they damage neurons and cause activation of microglia, astrocytes, and the complementary system. These processes are linked to high production of free radicals as well as an increased Ca^2+^ ion influx, which intensify neuronal apoptosis. Aβ can also stimulate the receptors for advanced glycation end products on the surface of neurons and induce the synthesis of proinflammatory molecules such as prostaglandins, excitotoxins, and cytokines, including tumor necrosis factor α (TNF-α). The resulting inflammation contributes to impaired neuronal function, ultimately leading to cellular death [1,13].

**Figure 2:**
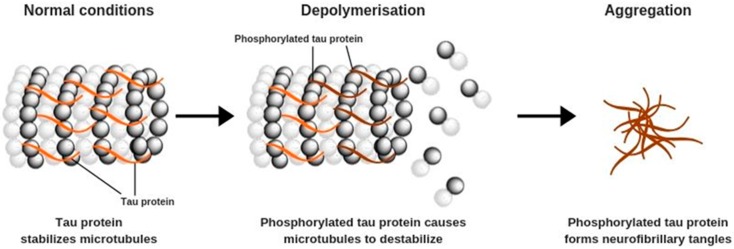
Hyperphosphorylation of tau protein.

Aβ formation is often accompanied by other pathological changes that occur primarily in pyramidal neurons and affect their structure. These processes are caused by increased phosphorylation of tau proteins, which begin to form polymers known as tau tangles (Figure 2). Under physiological conditions, tau proteins play an important role in the stabilization of microtubules, which are the structural elements of the cytoskeleton. Microtubules participate in cellular protein and enzyme transport that is needed for the correct functioning of neurons, including appropriate synaptic signaling [1,7,16].

Microtubules are fragile structures that require stabilization, which is provided by the interactions between their basic constituents, tubulins, and tau proteins. It is observed that increased tau phosphorylation causes disassembly of the microtubules and promotes the formation of tau tangles—a hallmark of AD. Cyclin-dependent kinase 5, one of the agents involved in this process, can be stimulated by the increased Ca^2+^ ion concentration in nerve cells, caused directly by Aβ aggregation. The result is depolymerization of microtubules, cytoskeleton deformation, disruption of the intracellular transport, and eventually, impaired functioning of the whole neuron. Tau tangles form toxic aggregates that activate the microglia and induce inflammation, leading to neuronal damage and cell death [7,16].

### 2.1. Neuronal Signaling Alteration and Changes in Brain Tissue

An important consequence of the loss of nerve cells is altered neurotransmission. AD is characterized by a significant reduction of acetylcholine content in the hippocampus, which is related to a lower level of the enzyme choline acetyltransferase necessary for its synthesis, which can be decreased by as much as 90%. Moreover, the apoptosis of noradrenergic and serotoninergic neurons results in a lowered concentration of noradrenaline and serotonin. Previous research indicated that levels of other neurotransmitters such as peptide Y and glutamic acid are also decreased. Impairment of synthesis of some hormones, mainly somatotropin and corticoliberin, is also observed [1,10,13].

Pathological processes occurring in neurons and impaired neurotransmission cause changes at the level of brain tissue. In AD, cell death is observed even in the most primordial structures such as the brainstem. Neuronal loss affects the locus coeruleus, which is responsible for sleep and fear regulation, as well as noradrenaline production; the raphe nuclei, where serotonin is produced, are also affected. Moreover, neuronal apoptosis occurs in the limbic system—neurons die both in the hypothalamus, which regulates an organism’s hormonal status, and the hippocampus, which is related to memory and cognition functions. Pathological changes include further brain regions, such as the nucleus basalis of Meynert in the substantia innominata, which forms a part of the cholinergic system, and the entorhinal cortex, where memory and smell-related processes occur. Loss of neurons can also affect other parts of the brain, with the damage expanding to the frontal, temporal, and parietal lobes, which are responsible for higher cognitive functions as well as movement, touch, and hearing processing [6,12,13,17]. Therefore, the neurodegeneration of brain regions that control somatic functions and produce neurotransmitters is directly correlated with the symptoms observed in patients with AD.

### 2.2. Genetic Factors

In most patients suffering from AD dementia, the causes of the disease are idiopathic. It remains unclear what roles genetic factors play in the late-onset type of AD. However, it has been proved that genetics is responsible for the heritable type of this disorder, which constitutes 1% of all AD cases. Early onset AD, which can begin even at the age of 30 years, has been linked to mutations in four genes: the genes encoding APP, presenilin 1, presenilin 2, and tau protein. It was observed that dysfunctional APP transcription correlates with the inherited AD occurrence [1,12]. Mutations in presenilin 1 and 2 genes can lead to an alternative APP cleavage, resulting in an increased production of Aβ. On the other hand, mutations in introns of the tau gene contribute to abnormal post-translational modifications of this protein and may promote overproduction of some of its isoforms. If these genetic factors are present, the development of AD is unavoidable. 

However, their role in late-onset AD is still controversial. One of the most important genetic factors influencing AD risk is *APOE* ε4 allele. The influence of the *APOE* gene has; however, been proven, that is, its ε4 isoform can increase Aβ accumulation. This protein is involved in cholesterol transport in the CNS and is responsible for binding lipoproteins to LDL receptors. It is synthesized in astrocytes and macrophages, and it is the most abundant apolipoprotein in the CNS. Research indicates that genetically determined changes in the amino acid sequence of *APOE* can lead to Aβ accumulation, exacerbate its neurotoxic effects, and increase neuroinflammation [4,18]. There are three different alleles encoding *APOE*: ε2, ε3, and ε4. The ε3 isoform is the most common one, while the ε2 isoform is rare, but it may be protective against AD. In contrast, having one copy of the ε4 isoform increases the risk of developing AD by 3-fold, while having two copies increases the risk by 12-fold. Additionally, unlike ε2 and ε3 isoforms, the ε4 isoform may contribute to earlier development of the disease, but it is worth noting that its presence does not lead to AD [19,20]. Furthermore, Aikawa et al. [21] have reported the ATP-binding cassette (ABC) reporter family role in the AD pathogenesis. ABC genes are involved in lipid metabolism through the regulation of phospholipids and cholesterol homeostasis. It seems that within ABC family the particular interest gene is the gene encoding ATP-binding cassette sub-family A member 7 (ABCA7) is related to an increased risk late-onset of AD. According to the in vivo and in vitro studies ABCA7 loss-of-function associate with AD development through multiple pathways and is linked with an increased Aβ formation. ABCA7 deficiency affects the phagocytic activity in macrophages and microglial Aβ clearance pathway, likely by facilitating endocytosis and/or processing of APP. Recent studies also suggest the importance of the sortilin gene (*SORT1*) that encodes a protein that binds APP and possibly prevents it from entering the endosomal-lysosomal pathway, thereby limiting Aβ formation [11,13,14].

### 2.3. Amyloid cascade hypothesis

Although there has been significant progress in the research explaining AD-related changes, many phenomena remain unclear. A widely accepted hypothesis describing AD development is the amyloid cascade. Its core mechanism is the accumulation of Aβ in the neuropile due to genetic defects in APP, environmental factors, and stressors such as toxins, which can be exacerbated by the presence of *APOE* ε4 isoform. Senile plaques trigger an immune response and induce inflammation, simultaneously increasing Ca^2+^ ion concentration in neurons, which contributes to tau hyperphosphorylation and its aggregation in the form of tangles [13,18,19]. The result is cytoskeleton disintegration, intracellular transport disruption, and neuronal damage. Consequently, neurons either degenerate or die, leading to the impairment of neurotransmission in many brain regions. These processes manifest themselves as dementia and other neurological symptoms. The current state of knowledge indicates that AD, like other chronic illnesses, is caused by more than one factor, including genetics and environmental stressors [13,20].

## 3. Alzheimer’s Disease: Risk Factors and Prevention

### 3.1. Unmodifiable and Modifiable Risk Factors

Some conditions and circumstances that influence the risk of developing AD are unavoidable, while other factors can be changed or even eliminated. The most important unmodifiable factor is age. Epidemiological data reveal that AD dementia occurs primarily in people over 65 years of age. Its risk increases from 3% at the age of 65 years to 47% in the population aged 84 years or more. Another important risk factor is sex. The lifetime risk of developing AD is twice as high in women as in men. Genetic predispositions also play an important role, as having one or more first-grade relatives suffering from AD significantly increases the risk of neurodegeneration. Presumably, the key factor is similar lifestyle, including physical activity level and diet, as well as inherited disadvantageous gene polymorphisms [15,20].

While age, sex, and genetics are unchangeable, the vast majority of risk factors for AD can be controlled or alleviated through a healthy lifestyle and avoidance of possibly harmful circumstances. Strong evidence indicates that traumatic brain injury is one of such conditions, especially when loss of consciousness and amnesia are involved. The most vulnerable groups are athletes and soldiers exposed to regular head injuries [22]. Epidemiological data suggest that education is also an important factor: the shorter the formal education period, the higher is the risk of developing dementia [23]. This correlation can be attributed to either not having intellectually demanding jobs or an inferior economic status of less educated people, both of which are linked to worse nutrition and lower health awareness [21].

Currently, much emphasis has been placed on the relationship between AD and chronic, lifestyle-dependent illnesses. A growing body of evidence indicates that cardiovascular dysfunction, especially hypertension, type 2 diabetes, and obesity induce neuronal changes that contribute to dementia [2,24]. In particular, epidemiological data indicate high frequently co-occurrence of diabetes and AD. Patients with type 2 diabetes have an increased risk of AD due to defects in glucose uptake in neurons. Likewise, glucose metabolism disorders are often observed among AD patients. The recent reports suggest an important role of caveolin-1 in this cross-link association as a possible linker of pathological changes in AD and type 2 diabetes. Caveolin-1 is an integral membrane protein of caveolae membranes involved in signaling cascades, lipid homeostasis and endocytosis. Moreover, caveolin-1 is involved in key AD pathological mechanisms such as APP metabolism and Aβ increased formation as well as tau hyperphosphorylation [25].

Thus, risk factors of noncommunicable diseases such as excessive stress, smoking, physical inactivity, and poor diet are thought to be at least partially responsible for neurodegeneration. It is estimated that a substantial percentage of all AD cases is caused by modifiable risk factors and could be prevented by efficient management of lifestyle changes [2,11,26].

### 3.2. Alzheimer’s Disease Prevention

Despite the constant progress in elucidating AD pathophysiology, efficient methods to prevent AD still do not exist. Research on human subjects indicates that in older populations, attempts to stop the already developed dementia yield unsatisfactory effects. The 2014 MAPT study consisting of nutritional advice, physical and mental exercise, and omega-3 supplementation showed that this type of multidomain intervention can be too late in people aged 75 years and over [27]. It seems that preventive actions should be taken earlier and include younger population, especially from dementia risk groups, who have not yet developed the disease. Although some medications have a positive impact on AD-related biomarkers, nonpharmacological preventive methods are catching researchers’ attention. Mental activity and social engagement are linked to a reduced risk of dementia, while bilingualism can delay AD onset. The results of the 2001 ACTIVE study showed that a special cognitive and memory training in people over 65 years of age significantly reduces the frequency of dementia and the effect of this training lasts even up to 10 years [28]. As chronic illnesses and AD risk factors converge, there is a hope that neurodegeneration can be stopped or attenuated by preventing the development of disorders such as hypertension, obesity, and type 2 diabetes. Strong evidence supports the crucial role of physical activity, which enhances nervous system functioning and reduces dementia risk by 50% [2,25]. In clinical trials, physical exercise has been proven to improve neuronal activity in the older population [11].

Moreover, observational studies reveal that a proper diet, as an underestimated factor, can provide neuroprotective effects. Higher consumption of fruits and vegetables, legumes (such as beans, peas, chickpeas, lentils), nuts, and fish, combined with lower intake of meat, high-fat dairy, and sweets, correlates with the reduced risk of AD [8,9]. In this regard, an especially well-studied nutrition pattern is the Mediterranean diet (MD). The meta-analysis of Psaltopoulou et al. [29] showed that this style of eating can prevent brain degeneration. Similar conclusions were obtained by Morris et al. [8,9] who aimed to compare the neuroprotective properties of MD, Dietary Approaches to Stop Hypertension (DASH) diet, and their combination called the MIND diet. They found that all three diets lowered the risk of AD; moreover, the MIND diet showed a significant effect even without the full commitment to the dietary rules [7,29].

Probably the most efficient approach to preventing AD is a combination of the aforementioned methods, that is, a holistic lifestyle improvement including mental activity, physical exercise, and proper nutrition. In this context, the 2014 long-term, randomized FINGER study, which involved 1200 older people from AD risk groups, yielded particularly promising results. The participants managed to maintain or even improve their cognitive functions as a result of a comprehensive program consisting of physical activity, cardiovascular health assessment and control, mental training, and a balanced diet rich in fruits, vegetables, whole grains, lean meat, low-fat dairy, and fish [30].

## 4. The Role of Bioactive Compounds

Proper nutrition is a key component of a healthy lifestyle, and it seems to play a crucial role in the prevention of neurodegenerative diseases, including AD. A balanced diet rich in bioactive compounds can reduce the risk of dementia [8,9]. Given the scarcity of human interventional studies it remains unclear whether these substances also exert all of the neuroprotective effects observed in in vitro and animal model studies in human under physiological conditions. There is also a lack of knowledge as to whether the amounts and chemical forms present in food make them sufficiently bioavailable. Nevertheless, their positive effects are largely supported by observational epidemiological cohort studies and experimental research that explain the molecular mechanisms of promising bioactive compounds action in the prevention of AD. These promising beneficial effects of selected bioactive compounds have been described in this review. Many of these compounds belong to one of the following chemical classes: phenolic compounds, fat-soluble vitamins and essential omega-3 fatty acids, isothiocyanates, or carotenoids.

### 4.1. Phenolic Compounds

Phenolic compounds are found in common plant foods, and one of their most important sources is olive oil, which contains oleuropein, hydroxytyrosol, and oleocanthal. Oleuropein is a glycosylated seco-iridoid with many beneficial properties; it has a strong antioxidant potential and protects nerve cells from neurotoxin-induced apoptosis [31]. It can also lower Aβ levels and prevent its aggregation, simultaneously reducing the expression of glutaminyl cyclase, an enzyme involved in Aβ synthesis. Moreover, oleuropein influences tau protein metabolism. In an in vitro experiment on Escherichia coli cell culture, Daccache et al. [32] demonstrated that oleuropein prevented the accumulation of a mutated, rapidly aggregating tau protein by 67% relative to the control group. For wild-type tau, the efficiency was 79%, while methylene blue, the reference tau aggregation inhibitor, showed 75% effectiveness. These results suggest that oleuropein can prevent the formation of toxic tau aggregates, probably due to the presence of aldehyde groups in the tautomeric forms of its aglycone metabolite.

In the digestive tract, oleuropein is hydrolyzed into another phenolic compound, hydroxytyrosol, which is also present in olive oil and has a higher bioavailability [31,32]. Hydroxytyrosol is a potent antioxidant and free radical scavenger, it can also activate phase II detoxification enzymes [33]. St-Laurent-Thibault et al. [34] showed in an in vitro study that it protects nerve cells against Aβ-induced toxicity. The authors suggested that hydroxytyrosol acts as an anti-inflammatory agent and reduces the activity of nuclear factor-kappa B (NF-κB) that triggers some of the neurotoxic reactions caused by amyloid plaques.

Another phenolic compound worth mentioning is oleocanthal, a substance responsible for olive oil’s bitter taste. It reduces inflammation by inhibiting the cyclooxygenase enzyme (COX), which participates in the synthesis of pro-inflammatory prostaglandins. Rodríguez-Morató et al. [31] showed its ability to reduce Aβ aggregation and modulate its clearance from the brain. In in vitro experiments conducted on E. coli-derived tau protein, Li et al. [35] showed that oleocanthal prevented the accumulation of the protein in comparison with the control group. Further research suggested that this compound forms covalent bonds with the amino acid lysine located on the C-terminus of the PHF6 peptide, a part of tau protein necessary for its polymerization [36]. These results indicate that oleocanthal can potentially ameliorate the pathological processes involved in AD development.

Observational studies indirectly confirm the neuroprotective effects of olive oil. Scarmeas et al. [36,37] showed that MD, which includes substantial amounts of fat source, is linked to a reduced risk of AD. The authors analyzed food frequency questionnaires collected from 1984 respondents, with an average age of 76 years, and found that higher compliance to the MD principles correlated significantly with lower AD risk, independently from other factors such as sex, education, BMI or *APOE* gene allele. Similar conclusions were drawn by Psaltopoulou et al. [29], who published a meta-analysis of 22 studies indicating that MD correlates with lower odds of developing AD and that even incomplete adherence to these dietary rules slows down the loss of cognitive functions. Researchers suggested that this protective effect may be related to the antioxidant and anti-inflammatory effects of the ingredients of MD. They also claimed that this type of diet can be efficient in preventing CNS degeneration.

The aforementioned benefits of olive oil were also corroborated in a randomized, controlled trial conducted by Valls-Pedret et al. [38]. The 4-year-long dietary intervention involved 447 older people from an AD risk group. They were divided into 3 subgroups: the first one followed the MD supplemented with 1 L/week of olive oil, the second one followed the MD supplemented with 30 g of nuts per day, and the third one followed a low-fat diet. Changes in cognitive functions and memory were assessed through 6 specific tests conducted at the beginning and end of the study. The participants taking MD supplemented with olive oil showed improvements in their working memory and attention and scored better in the Mini-Mental State Examination (MMSE), a dementia level assessment test.

Other neuroprotective phenolic compounds are anthocyanins. They belong to the flavonoid group and are responsible for red, violet, and blue color of many fruits and vegetables. According to Li et al. [39], anthocyanins ameliorate oxidative stress by lowering free radical production and lipid peroxidation. They also reduce prostaglandin synthesis by inhibiting COX. Furthermore, anthocyanins increase the activation of the FKBP52 protein, which has an affinity for phosphorylated tau protein and prevents its aggregation. They lower the intracellular Ca2+ ion concentration and inhibit caspase-3, which regulates neuronal apoptosis. Neuroprotective effects of anthocyanins were analyzed in vitro by Yamakawa et al. [40], who found that two of these substances, namely delphinidin and cyanidin, complexed with Aβ peptides to inhibit their aggregation. The authors confirmed these observations in their next experiment, in which mouse neurons were exposed to both Aβ and anthocyanins. The survival was significantly higher among cultures treated with solutions containing delphinidin or cyanidin than in cells exposed to only toxic peptides. This suggests that anthocyanins may neutralize the toxic effects of amyloid and protect nerve cells.

The beneficial effect of anthocyanins was also confirmed in animal models. Gutierres et al. [41] studied the effects of anthocyanins in rats with AD induced by injection of streptozotocin into the cerebrospinal fluid. Before the drug was administered, the animals had been given orally a formulation containing grape skin-derived anthocyanins for 7 days. The dietary intervention prevented streptozotocin-induced increase in acetylcholinesterase activity, which is the enzyme responsible for acetylcholine degradation. It also protected against memory loss as assessed by behavioral tests and measurement of anxiety, memory, and motor functions. In addition, anthocyanins inhibited excessive synthesis of nitrogen reactive species in the cerebral cortex and hippocampus [41].

One of the best sources of anthocyanins is berries. An observational study by Devore et al. [42], which involved 16,010 women aged 70 years or older, showed that higher consumption of blueberries and strawberries was linked to a delayed loss of cognitive function. The total intake of all flavonoids and anthocyanins, calculated on the basis of food frequency questionnaires, was related to a slower deterioration of mental abilities, as assessed by validated phone interviews. Neuroprotective effects of bioactive compounds present in berries were also confirmed in experimental trials. A study by Krikorian et al. [43] involved nine older persons with an average age of 76 years, who suffered memory impairment. Daily intake of phenolic compounds was maintained between 6 to 9 mL/kg by using a dosing schedule determined by body weight. Individuals weighing 54–64 kg obtain 444 mL of blueberry juice per day, those weighing 65–76 kg consumed 532 mL/day, and those weighing between 77–91 kg consumed 621 mL/day. For 12 weeks, they supplemented their standard diet with either a berry juice rich in anthocyanins or a placebo. Their learning abilities and memory were assessed verbally at the beginning and end of the intervention. The researchers found that the consumption of berry juice improved mental condition of the participants and, additionally, ameliorated depression symptoms as well as lowered fasting glucose levels.

Curcumin, a natural ingredient of turmeric, is another phenolic compound that shows promising neuroprotective properties. It is a potent antioxidant, reduces protein oxidation products, attenuates inflammation by inhibiting both COX and lipoxygenase enzymes, and lowers microglia activity. The chemical structure of curcumin is similar to Congo red, a substance used for staining senile plaques. This characteristic makes it efficient in binding Aβ and preventing its oligomer formation [44]. Observational studies suggest that curry containing a high amount of curcumin can promote the maintenance of mental functions. Ng et al. [45] juxtaposed the intake of this condiment and the results of MMSE tests conducted on a population of 1010 Singapore inhabitants aged 60–93 years. Compared to persons using curry “never or rarely,” the participants who claimed to consume it “occasionally” and “often to very often” scored significantly better in cognitive tests. The influence of curcumin on mental functions was also analyzed by Small et al. [46] in a randomized, double-blind, placebo-controlled trial that involved 40 persons aged 51–84 years. The participants in the intervention group took a curcumin supplement (90 mg curcumin per day) twice a day for 18 months, while the control group received a placebo. Both at the beginning and end of the study, their cognitive functions were assessed through attention, verbal, and visual memory tests. Thirty participants were also scanned with positron emission tomography to determine the effect of curcumin on tau protein and Aβ accumulation in the brain. Compared to the placebo group, the intervention group showed significant improvement in attention and memory. The intervention group also showed decreased aggregation of Aβ and tau protein in the amygdala and hippocampus, which indicates a strong neuroprotective effect of curcumin [46].

Genistein, an isoflavone found mainly in soy products, is another compound that is potentially effective in preventing AD. It reduces oxidative stress by inhibiting the synthesis of oxygen reactive species. Moreover, genistein protects mitochondria by increasing reduced-to-oxidized glutathione ratio and lowering 8-oxo-2′-deoxyguanosine, a marker of mitochondrial DNA damage [47]. It also prevents apoptosis by restricting caspase-3 activity and ameliorates inflammation through reduction in TNF-α and NF-κB levels. Genistein can act as a β-secretase inhibitor and an α-secretase promotor, thus decreasing Aβ synthesis and senile plaque formation [47]. Ye et al. [48] conducted an experiment on an animal model, in which rats were administered genistein for 7 days and then received an intracerebral injection of Aβ to emulate AD-like conditions. The authors found that the pre-injection treatment reduced neuronal damage and lowered phosphorylated tau levels in the hippocampus. Behavioral tests showed that animals from the intervention group exhibited improvements in their memory and learning abilities as compared to rats that did not receive genistein. The neuroprotective properties of this isoflavone are supported by observations of Asian populations, whose traditional diets contain large amounts of soy products. Previous studies indicate that high intake of these foods can protect against dementia. Ozawa et al. [49] analyzed nutrition data compiled over 15 years from 1006 older Japanese individuals aged 60–79 years. The authors concluded that a diet richer in vegetables, algae, dairy, and soy correlated with a lower risk of dementia. Among persons adhering most strictly to this nutrition model, the risk was reduced by two-third, which suggests that diets high in soy products containing genistein could protect the CNS [49]. 

Among other promising inhibitors Aβ synthesis, the interesting neuroprotective effect of Hop-extracts was observed. Hop-extracts have been reported to reduce Aβ production in cultured cells due to inhibition of γ-secretase activity, and prevent learning and memory impairment as well as Aβ depositions in mice [50]. Sasaoka et al. [50] purified a major active component in Hop extracts, Garcinielliptone originally isolated from Garcinia, that inhibits γ-secretase and suggested that Garcinia extracts might also have the potential to reduce Aβ production or accumulation.

### 4.2. Omega-3 Fatty Acids and Fat-Soluble Vitamins

Bioactive compounds potentially useful in AD prevention can also be found among vitamins and essential fatty acids, which are exogenous substances necessary for the proper functioning of the human body. Among various tissues in the body, the CNS is particularly vulnerable to oxidative stress due to its high oxygen use and high content of polyunsaturated fatty. Suppressing oxidative stress by lipophilic antioxidants and vitamins is considered to be a beneficial strategy in AD risk reduction. One of these substances is docosahexaenoic acid (DHA), a polyunsaturated fatty acid present mainly in fish. It belongs to the long-chain omega-3 family, which also includes eicosapentaenoic acid (EPA) and n-3 docosapentaenoic acid (DPA). The most abundant brain n-3 fatty acid is DHA [51,52]. In comparison to EPA, DHA content in the brain is much higher. As a vital structural membrane phospholipids component of the brain cells, DHA is present in the cerebral cortex and synaptic membrane regions. Additionally, Δ4 desaturase activity, the enzyme that is involved in DHA synthesis, decreases with age, which results in a decrease in DHA synthesis in the elderly. These features make DHA the most promising omega-3 fatty acid in the context of age-related CNS diseases. DHA brain concentration depends on dietary intake, as well as on liver conversion from its shorter chain precursors. DHA nutritionally essential PUFA precursors: EPA and α-linolenic acid (ALA) are present in the brain in very small amounts [53]. Unlike DHA, concentrations of which are around 10,000 nmol/g of brain, EPA is commonly reported to be several orders of magnitude lower (less than 250 nmol/g) in the whole brain of both humans and rodents due to its rapid and extensive β-oxidation. Brain EPA levels are regulated rather by β-oxidation and rapid metabolism, but not by uptake by neurons and glial cells [54]. DHA competes with arachidonic acid, a precursor of prostaglandins, for embedding in the cellular membrane. Therefore, it can lower brain inflammation by reducing the synthesis of these eicosanoids. Docosahexaenoic acid also affects APP metabolism by increasing the levels of chaperons, which participate in APP folding and prevent Aβ formation. DHA can protect the nervous system through increased synthesis of neuroprotectin D1 and brain-derived neurotrophic factor (BDNF), both of which impede neuronal damage and participate in neurogenesis [51,55]. Furthermore, DHA influences Aβ and tau aggregation, which was confirmed by Green et al. [56]. The authors conducted an in vivo experiment on transgenic mice producing both Aβ plaques and tau tangles. The animals were assigned to either a control group or one of three intervention groups receiving DHA, DHA and arachidonic acid, or DHA and n-6 docosapentaenoic acid for 3, 6, or 9 months. The authors found that diets supplemented with DHA inhibited Aβ accumulation and lowered tau protein levels after 3 months. After 9 months, this effect was even stronger, especially in the group administered only DHA, which additionally showed reduced levels of phosphorylated tau [56].

Epidemiological data indicate that DHA can play an important role in neurodegenerative disorder prevention. Zhang et al. [57] published a meta-analysis of 21 cohort studies, which involved 181580 participants and lasted from 2 to 20 years. They showed a correlation between fish consumption and a reduced risk of cognitive decline. While they found no association between EPA intake and the risk of AD, the increase in dietary DHA by only 0.1 g/day was linked to lower dementia and AD risk [57]. Experimental studies yielded similar results. In a randomized, double-blind clinical trial that involved 33 AD patients who received either placebo or omega-3 preparation, Levi et al. [51] observed a link between cerebrospinal fluid DHA level and lowered markers of tau phosphorylation. At the same time, higher DHA concentrations showed a direct correlation with an increased expression of interleukin-1 receptor type II, which has anti-inflammatory properties. These results indicate that DHA may counteract neurodegeneration underlying processes.

Vitamin D is another neuroprotective compound. Even though its synthesis is stimulated primarily by ultraviolet radiation, it can also be obtained from dietary sources such as fish [58]. Vitamin D can be considered as a dementia-preventing agent because of its anti-inflammatory and anti-amyloid properties, such as enhanced Aβ clearance from the brain, through phagocytosis but also have an impact of Aβ production and enzymatic degradation [59]. It inhibits TNF-α and interleukin-6 production in the microglia as well as reduces Aβ levels in the hippocampus and promotes its phagocytosis by macrophages. Vitamin D also influences calcium homeostasis, which is often disturbed in neurodegenerative diseases [59,60]. Morello et al. [61] investigated the effects of this vitamin on mice expressing human APP. The animals were fed either a diet without vitamin D, a diet providing 1000 IU/kg of vitamin D, or a diet with 7500 IU/kg of vitamin D. One group of mice underwent this intervention for 6 months from the beginning of the experiment, while the other group underwent it from the 4th to the 9th month from the beginning of the experiment. The study showed that vitamin D supplementation improved memory among animals receiving it in the initial stage of the disease. Additionally, the authors concluded that the highest dose significantly influenced neurogenesis by promoting neuronal growth in the hippocampus [61]. Important data regarding cognitive effects of vitamin D were provided by Littlejohns et al. [62], who tracked a group of 1658 persons for 5 years and measured their blood concentration of 25(OH)D3 both at the beginning and end of the observation period. The authors confirmed the initial hypothesis that low levels of vitamin D correlate with significantly higher risk of AD. Similar results were obtained by Feart et al. [63], who monitored cognitive functions of 916 subjects over 65 years for 12 years. The authors measured vitamin D levels of the participants and assessed their mental abilities with 5 different psychological tools. Statistical analysis showed that compared to persons with normal vitamin D blood concentration, those who showed deficiency in this nutrient showed a higher rate of cognitive decline and had a 3-fold higher risk of AD [63]. Annweiler et al. [64] also confirmed the neuroprotective role of vitamin D in an intervention study that involved 44 patients with an average age of 81 years. The authors found that 2 years of vitamin D supplementation (either 800 IU per day or 100000 IU per month) significantly improved cognitive test results. Furthermore, better scores in behavioral tests correlated with higher blood vitamin D concentrations, which shows the importance of this compound in preserving mental health of the elderly at risk of developing dementia.

Likewise, AD prevention can be aided using vitamin E. It includes eight substances, namely four tocopherols and four tocotrienols, either of which can occur in one of the following four chemical forms: α, β, γ, or δ. They can be found mainly in nuts, seeds, and vegetable oils [65]. Vitamin E is a potent antioxidant present in brain tissue. It lowers lipid susceptibility to oxidation by as much as 60%, protects cellular membranes and DNA from free radicals, and prevents decrease in glutathione and catalase levels, which is one of the features of AD. Vitamin E can also act as an anti-inflammatory agent by inhibiting COX and lowering NF-κB activity [65,66,67]. Observational studies suggest that the antioxidant properties of vitamin E can protect against dementia. Cherubini et al. [68] analyzed the relationship between blood levels of vitamin E and mental health among 1033 subjects aged over 65 years. The authors found that compared to the highest concentrations, lower levels of vitamin E predicted a greater risk of developing dementia. Similar results were obtained by Mangialasche et al. [69], who observed a population of 232 persons aged over 80 years for 2 years. The authors measured participants’ blood concentrations of all 8 vitamin E forms and conducted the MMSE test. They concluded that high levels of tocopherols and tocotrienols lowered the risk of dementia by as much as 45%.

Beneficial effects of vitamin E were also indirectly confirmed in the aforementioned randomized clinical trial by Valls-Pedret et al. [38]. In this study, the MD supplemented with nuts rich in vitamin E improved memory and slowed down cognitive impairment in older subjects with AD risk. These results are supported by another randomized clinical trial conducted by Dysken et al. [70], which involved 613 patients with mild to moderate AD. The participants were divided into 4 groups who received either the drug memantine (moderate-affinity NMDA antagonist) α-tocopherol (2000 IU/day), memantine with α-tocopherol, or placebo. At the end of the 2-year intervention, patients supplemented with vitamin E experienced a significantly slower rate of cognitive decline and, compared to the placebo group, a delay of 6.2 months in AD development.

### 4.3. Isothiocyanates

The next group of bioactive compounds potentially helpful in AD prevention is isothiocyanates. They are derivatives of glucosinolates present primarily in cruciferous vegetables. Because of the sulfur atom in their molecule, isothiocyanates act as antioxidants, especially those containing an aromatic ring directly bonded to the thiocyanate group. They are also strong COX inhibitors and show anti-inflammatory effects. Moreover, some isothiocyanates efficiently suppress acetylcholinesterase activity, thereby prolonging the half-life of acetylcholine, a neurotransmitter whose concentration is usually lowered in patients with AD [71]. One of the most researched isothiocyanates is sulforaphane, a compound derived from glucoraphanin during the mechanical processing of plant tissues. It increases the levels of antioxidant enzymes such as glutathione peroxidase and glutaredoxin. In both neurons and glial cells, it increases the activity of sulfiredoxin, which regenerates other antioxidant enzymes. Furthermore, in nerve cells, sulforaphane modulates the activity of proteasomes, which are enzymatic complexes containing high amounts of proteases and are potentially helpful in Aβ clearance [72].

Epidemiological studies suggest that isothiocyanates could show neuroprotective effects. Nurk et al. [73] analyzed the diet and mental functions of 2031 persons aged 70–74 years. The authors found that those who ate more cruciferous vegetables such as cabbage, cauliflower, broccoli, and Brussels sprouts scored better in cognitive tests. Experimental studies also showed promising results. Lee et al. [74] investigated the effect of sulforaphane on a transgenic mouse model of AD; for 8 weeks, the animals were given an oral gavage infusion of either one of two doses of the isothiocyanate or a saline solution. Sulforaphane administration lowered Aβ and tau levels in the hippocampus—an effect linked to an increased expression of the CHIP protein, which accelerates the degradation of amyloid peptides and prevents the formation of tau tangles. Mice from the intervention group also lost memory and learning abilities to a lesser degree than the other animals. These results indicate that sulforaphane affects the main AD pathological mechanisms and could potentially provide neuroprotective benefits.

### 4.4. Carotenoids

Carotenoids are plant-derived pigments present in many fruits and vegetables, which are responsible for their yellow, orange, and red colors. They can also be produced by microalgae, a food source for marine animals, which makes these algae an additional dietary source of these compounds. Carotenoids play an auxiliary role in photosynthesis and protect against photooxidation. One of the most beneficial carotenoid compounds is astaxanthin. It acts as a free radical scavenger and reduces oxidative stress, lipid peroxidation, and protein peroxidation products. It also increases the levels of antioxidant enzymes such as catalase and superoxide dismutase. Astaxanthin protects nerve cells against apoptosis by lowering caspase-3 activity and increases neurogenesis by influencing mitogen-activated kinases [75]. Neuroprotective properties of astaxanthin were confirmed by Katagiri et al. [76] in a randomized, double-blind clinical trial that involved participants aged between 45 and 64 years, who received an extract rich in astaxanthin (6 or 12 mg astaxanthin/day) for 12 weeks. The authors found that compared to the placebo group, the both intervention group showed better scores in cognitive and learning tests [76].

Mental benefits can also be provided by two other substances from this group: lutein and zeaxanthin. An observational study by Christensen et al. [77], which included 2796 subjects over 60 years old, showed a link between higher consumption of these carotenoids and better cognitive functions. Lutein and zeaxanthin intake was determined by 24-hour recall, and the participants performed three tests that measured their memory, verbal fluidity, and attention. The authors concluded that a higher intake of carotenoids, both from food and supplements, correlated significantly with better mental performance. These positive effects were confirmed in a randomized, double-blind placebo-controlled study by Power et al. [78]. This study involved 91 persons with an average age of 45 years, who received either a supplement containing lutein (10 mg), zeaxanthin (2 mg), and meso-zeaxanthin (10 mg) or placebo for 12 months. Several neuropsychological tests showed that the participants ingesting additional carotenoids showed improvements in their cognitive functions and had better episodic memory than the placebo group. The authors suggested that these results can be due to the antioxidant action of lutein and zeaxanthin and their positive effect on neuronal membrane integrity.

Lycopene, another carotenoid compound, also has neuroprotective potential. It is a potent antioxidant that effectively neutralizes singlet oxygen, lowers lipid oxidation markers, and protects DNA against oxidative damage [79]. An in vitro study by Hwang et al. [80], which was performed on human neuronal cultures exposed to lycopene and subsequently to Aβ, showed an increased survival rate and decreased apoptosis in cells treated with lycopene. The carotenoid also lowered free radical level and prevented amyloid-induced mitochondrial dysfunction [80]. These outcomes indicate that lycopene could help in preventing neurodegenerative processes. 

### 4.5. MIND Diet

Although the exact relationship between diet and AD development is not fully understood and more experimental human studies are required, epidemiological data seem promising. It was shown that both the MD and DASH diets promote mental health, but an intervention that combines their selected neuroprotective components could be even more effective [8,9]. This kind of nutrition system, called the MIND diet, was reported by Morris et al. [8,9] and is based on whole foods rich in bioactive compounds. Its primary components include the following: whole grains (at least 3 servings/day), green leafy vegetables (at least 6 servings/week), other vegetables (at least 1 serving/day), berries (at least 2 servings/week), fish (at least 1 serving/week), poultry (at least 2 servings/week), legumes (more than 3 servings/week), nuts (at least 5 servings/week), and olive oil as the main added fat source and wine (1 serving/day). The effectiveness of the MIND diet was confirmed in a 4.5-year observational study on 923 persons aged between 58 and 98 years, which showed a decreased risk of AD by as much as 53% in participants adhering to this nutritional strategy [8,9]. Even though further clinical trials on the effectiveness of the MIND diet are necessary, the current state of knowledge suggests that food rich in bioactive compounds can contribute to AD prevention.

## 5. Conclusions

**Figure 3:**
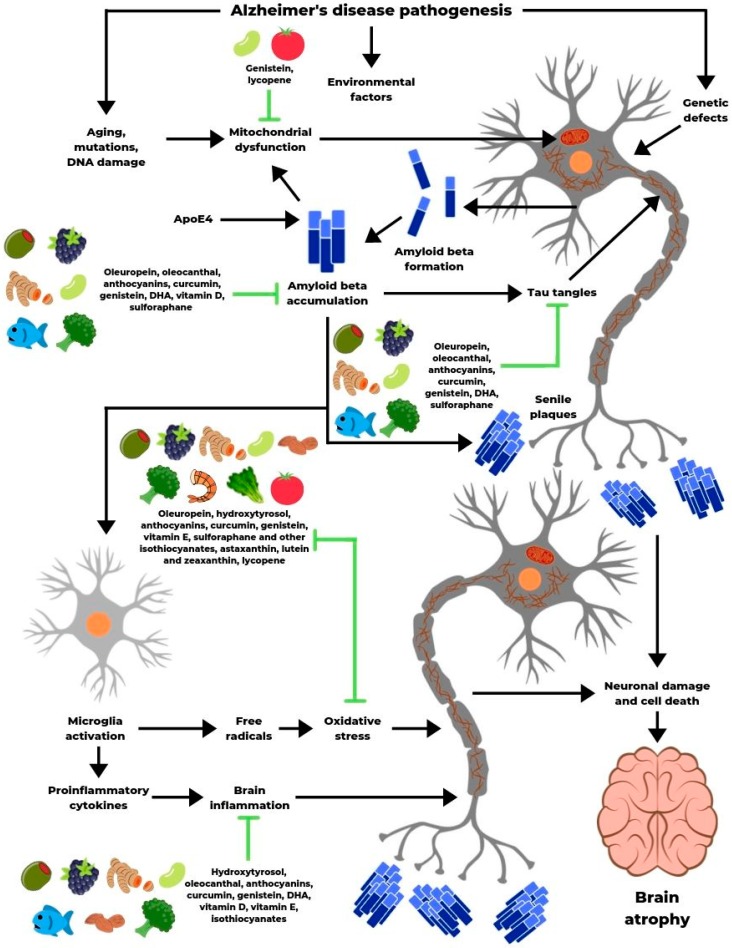
Influence of bioactive compounds on the pathogenesis of Alzheimer’s disease.

Both observational and experimental studies indicate that bioactive compounds in food influence the underlying pathological processes characteristic of AD (Figure 3). They can also improve cognitive functions. Their mechanisms of action are diverse, but the primary beneficial effects include the following:

- reduction of Aβ levels and tau phosphorylation rate
- prevention of Aβ and tau aggregation
- defense against oxidative stress
- anti-inflammatory activity
- protection of cellular structures and inhibition of neuronal apoptosis

Because of their beneficial effects, bioactive food compounds can constitute an important part of AD prevention and treatment. However, there is still insufficient data regarding their optimal doses, bioavailability, differences between chemical forms, and possible interactions with other dietary components. Although more research in this area is necessary, observational studies show that intake of food products rich in these substances provides indisputable benefits. Nutrition models such as the MD, the DASH diet and, especially, the MIND diet, provide high amounts of bioactive compounds and reduce the risk of AD.
